# Guardians of Public Value: How Public Organizations Become and Remain Institutions

**DOI:** 10.1007/978-3-030-51701-4_1

**Published:** 2020-11-13

**Authors:** Arjen Boin, Lauren A. Fahy, Paul ‘t Hart

**Affiliations:** 1 Department of Political Science, Leiden University, Leiden, The Netherlands; 2 School of Governance, Utrecht University, Utrecht, The Netherlands; 3 School of Governance, Utrecht University, Utrecht, The Netherlands; 4 Department of Political Science, Leiden University, Leiden, The Netherlands; 5 School of Governance, Utrecht University, Utrecht, The Netherlands; 6 School of Governance, Utrecht University, Utrecht, The Netherlands

## Abstract

*It’s an institution*—a phrase we have all come across or may have used. We intuitively understand what it means. There is something special, perhaps mythical, about them. We value these institutions. We may even find it hard to imagine a life without some of these institutions. In this chapter, we offer a definition of institutions and introduce our theoretical framework (based on the work of Philip Selznick). We introduce the case studies in this book and identify patterns of institution building.

## Institutions as Enigmas

*It’s an institution*—a phrase we have all come across or may have used. We intuitively understand what it means. The Louvre is not just a museum. Ascot is not just a horse race. The Ryman Auditorium in Nashville is not just a music venue. Wembley is not just a stadium. Cambridge University is not just a university. These are *institutions*. There is something special, perhaps mythical, about them. We value these institutions. We may even find it hard to imagine a life without some of these institutions.

Some public organizations, too, have achieved this special ‘institutional’ status: organizations that—in the words of Philip Selznick (1957: 17), the pioneering scholar of institutions—have become ‘infused with value beyond the technical requirements of the task at hand’. Institutions embody and safeguard certain values that are important to a society (Hendriks 2014). Institutions guard these values against overt attacks and the forces of erosion.

The average citizen may never wonder about the critical importance that these public institutions play in their lives. At the same time, academics hardly ever question the importance of institutions. It is simply assumed. This combination of limited public interest and academic conventional wisdom has done little to further research into the way institutions emerge and persevere. In their efforts to protect their institutions, leaders cannot fall back on a full body of academic research findings.

Such protection is increasingly necessary. Government agencies as well as other public sector organizations today face a climate where performance expectations are relentless, transparency and accountability regimes have thickened, and there is little tolerance for failure. Critical factors in the broader environment—technology, economic tides, societal beliefs and values, political fault lines and ‘rules of the game’— change constantly, sometimes rapidly and deeply. No institution, however powerful and well-regarded, is immune to ‘events’ and to the churning tides of public opinion. Even long-standing institutions face reputational and sometimes existential crises.

Yet, even in this volatile environment, some public organizations remain deeply valued by the public. They have not just survived challenges and controversies; they have found ways to thrive. They have adapted in the face of crises, preserving their institutional character while meeting newly imposed demands. They have become iconic features of the public landscape. That’s why we call them public institutions.

This volume is about these public institutions. We have selected twelve organizations that have met Selznick’s definition for at least a significant part of their lifespan. We examine each of these twelve institutions in some depth to understand their nature, formula and impact. We seek to show what scholars and organizational leaders can learn from them, warts and all. The overarching puzzle that drives the case studies collected here is simple: *Why do some public organizations develop into institutions, proving remarkably adept at becoming and remaining publicly valued over relatively long periods of time?*

In this introductory chapter, we explain how in Selznick’s work and of those that have followed in his footsteps institutions differ from organizations. We then describe the key conceptual and analytical tools that have informed the case studies in this volume, and briefly introduce each of the cases. Next, we present a thematic preview of the institutional patterns that emerge when we look across the twelve case studies. We offer these patterns as pointers for classroom discussion, but also as starting points for more empirically informed theorizing about how and why public organizations become institutions (and how they can also ‘deinstitutionalize’). Finally, we ask a pertinent and perhaps uncomfortable question: can public organizations that effectively and authoritatively guard public value and receive widespread recognition for doing so, continue to flourish in our turbulent and more unforgiving age?

## How Do We Know an Institution When We See One?

We use the concept of institution to describe a particular category of organizations. An organization is, in essence, nothing more than an established way of cooperation between two or more individuals (Barnard 1938). What sets an organization apart as an institution is its pursuit of aims that are widely considered to fulfil a societal need, its reliable performance over time, and its exemplary conduct as perceived by societal constituencies. The cases in the book provide powerful illustrations of these institutional characteristics:

- The *BBC* has been producing a judicious and widely respected mix of news and entertainment, has built itself into a global media brand while adapting successfully to major technological (such as satellite and online television) and regulatory changes (introduction of commercial broadcasting).
- The scientific centre for particle physics research *CERN* has gained international recognition as the hub in its field, has kept on pushing the boundaries of knowledge, has educated generations of influential researchers and has sparked the public’s imagination both through its mammoth underground research facility (the Large Hadron collider) and its discoveries (the Higgs Boson particle) as well as through its spinoff technologies (such as the World Wide Web).
- *Médicins Sans Frontières* (Doctors Without Borders) has been much acclaimed for its fearless commitment to providing medical care to populations caught in complex and devastating conflicts around the world. It has become a beacon of courageous behaviour in very challenging and dangerous circumstances. Moreover, it has repeatedly called attention to the follies and excesses of the humanitarian aid industry, and in doing so has become its moral conscience.

These examples suggest that there is something special about institutions: they are regarded as more valuable than just any organization. Goodsell (2011a: 477) refers to *mission mystique*, which he defines as an organization’s ‘aura of positive institutional charisma that is derived from the nature of its mission and how well it is carried out’. Aura is, of course, a matter of perception: people must recognize something special in what an organization does and how it performs its tasks. This subjective dimension of institutions makes it challenging for social scientists to arrive at a more systematic way of establishing why and to what extent an organization can be categorized as an institution. But it is a challenge that has to be met.

**Fig. 1.1:**
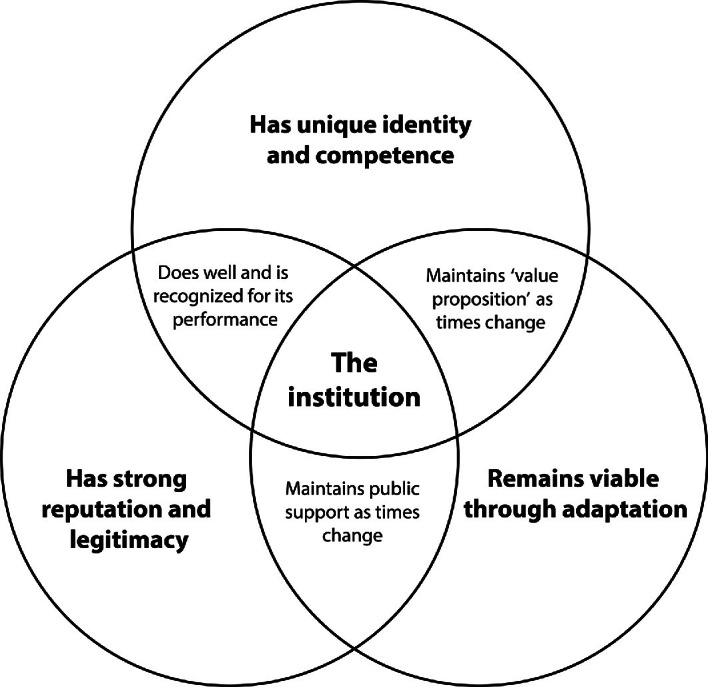
An organization as institution: Selznick’s criteria

 Selznick’s (1957) classic distinction between organizations and institutions provides a helpful tool in this endeavour. He formulated three criteria that can help us identify the institution in a population of organizations (see Fig. 1.1). Selznick’s framework can also help us track and interpret institutional trajectories: how an organization takes on institutional characteristics and how an institution may deinstitutionalize. Let’s have a look at these three criteria.

Distinct identity and unique competence . An institution has a clearly developed and widely recognized identity that communicates to both its members and the outside world what it seeks to achieve and why, what the dominant practices in the organization are, and how it addresses conflicts that occur in the pursuit of its aims. Mark Moore (1995)—a self-confessed Selznickian—speaks about identity in terms of a ‘value proposition’ and refers to institutional competence as the ‘operational capacity’ of an organization. An institution’s identity and competences are well-suited to meet societal aspirations and expectations. An institution has fostered a strong alignment between the rationale for its existence and the day-to-day strategies and practices it deploys. This alignment is routinely reconfirmed in the responses that institutional actions draw from its audiences.

Strong reputation, high legitimacy . An institution is trusted and respected, to such a degree that its existence is sometimes taken for granted. Employees are proud to work there and intrinsically motivated to contribute to the cause. The institution’s external stakeholders— Moore (1995) speaks of an ‘ authorizing environment’— support the institution through thick and thin by what they say and do. They provide funding, procure its products and services. They trust it to do the right thing in the right manner. They forgive its occasional lapses, to a much greater extent than they would for an organization not endowed with *mission mystique*. It is hard to imagine that anyone would even propose to abolish it.

Enduring viability through adaptation . An institution has adaptive capacity, which helps it to stand the test of time. This is not just about changing structures and practices to make the organization more effective or efficient. Institutions have the paradoxical ability to change in order to remain the same—changing whatever must be changed to protect the institutional core (Ansell et al. 2015). An institution can consistently deliver on its mission, working in ways that reaffirm its value proposition and satisfy the evolving expectations and norms of its stakeholders. It does what most public organizations find really hard: adopting and implementing reforms that prove to be effective.

An important recent study that provides support for much of what Selznick was proposing, albeit cast in slightly different language, is that of Charles Goodsell (2011a, b), who examined the organizational history and development of ‘ mission-driven’ public agencies in the United States, including such iconic institutions as NASA and the National Forest Service. He provides an in-depth, case-oriented study of what life in public institutions looks and feels like. Table 1.1 gives us Goodsell’s matrix of cultural characteristics and organizational practices that his institutions all shared. It provides a useful elaboration of Selznick’s institutional characteristics.

**Table 1.1:** Organizational features of institutionalizing public organizations

	Prime qualities	Essential elaborations	Temporal aspects
*A purposive aura*	A central mission purpose permeates the agency	The societal need met by the mission is seen as urgent	Has a distinctive reputation based on achievement
*Internal commitment*	Agency personnel are intrinsically motivated	Agency culture institutionalizes the belief system	Agency history is known and celebrated
*Sustaining features*	Beliefs are open to contestation and opposition	Agency enjoys qualified policy autonomy to permit appropriate adaptation	Agency renewal and learning are ongoing

*Source* adapted from Goodsell (2011a: 480)

The combination of Selznick’s and Goodsell’s institutional characteristics allow us to make a snapshot of any organization in order to determine whether, or to what extent, it qualifies as an institution. Importantly, the three criteria can be applied in a dynamic manner: we can ‘shoot’ a film of the institution’s development by applying the criteria at several points in time. That film would show the ebbs and flows of an organization’s institutional status, identifying periods of strong institutionalization but also periods of institutional decline.

Such a dynamic perspective on institutional development is crucial. Institutions are never born as ‘institutions’, though their architects and foundational leaders may have high hopes for them. They *become* institutions. They see the light as a small social group, a budding network, a small organization; some develop and gain institutional characteristics. We refer to this as a process of institutionalization (Boin and Goodin 2007).

But just as an organization can take on institutional characteristics, an institution can also *lose* institutional characteristics. Institutions can *de*institutionalize (Oliver 1992; Suchman 1995; Boin and ‘t Hart 2000; Boin 2001). Its mission can become less relevant, or diluted by *mission creep*. Mission creep refers to the widening of the mission, adopting new ambitions and tasks that distract from the original aims. Also, the organizational structure, culture and established practices may lose relevance, hindering rather than helping to achieve the mission. The institution can become ridden with internal conflict, or lose touch with its authorizing environment. 

## Institutions as Guardians of Public Value

 Institutionalization brings enormous benefits for public organizations. It helps to bind members to a common cause, thus diminishing the transaction costs in the organization. It buttresses against the winds of fashion, as the high level of legitimacy effectively grants a degree of autonomy so that leaders of an institution can chart its course. Institutions inspire confidence in those to whom they are accountable and as a result are less scrutinized than other organizations. When an institution is found to have failed or strayed, it is forgiven for more and for longer than organizations that lack their charismatic aura. Institutions are, in other words, better prepared to weather the storms of failure, scandal and crisis that any organization faces in its lifetime—*provided*, as Selnick (1957) reminds us, that they remain responsive and adaptive to the environments they work in and from which they derive their public licence to operate (in fact, Selznick identified this as the most difficult leadership task).

Institutions also benefit society. They fulfil certain functions in ways that are appreciated by that society. As the case studies in this book will show, these functions can vary widely. The institutions discussed in this book:

- Provide fair elections
- Protect against corruption
- Offer a trusted source of news
- Preserve the value of money
- Create a legal framework that benefits collaboration
- Preserve cultural traditions
- Protect the integrity of sports
- Create conditions for path-breaking research
- Protect a society against disaster
- Assist helpless victims of disaster
- Foster a shared interpretation of complex research findings.

A society needs institutions to ensure that we will have fair elections in the future, that we may expect a continued stream of validated news, that we can trust research findings, and be confident that future disasters will not cut the lives of citizens short. But institutions do more than fulfilling useful functions. They guard against the erosion of these functions and protect the values that underlie them. Institutions are the guardians of a state’s promises; they preserve a society’s hopes and ambitions.

Our fascination with institutions builds on two empirical observations. First, it has been observed that a minority of public organizations live a long life. A majority perishes (a sizeable chunk does not even make it longer than a decade) (Lewis 2002). Second, only a handful of those survivors meets the institutional test set forth by Selznick. We therefore conclude that *public institutions are exceptions or outliers.* We want to get to know these outliers.

## Are Institutions ‘Good’ by Definition?

 A key challenge for institutional scholars is dealing with the normative connotations that come with the “institution” concept. We generally reserve the term ‘institution’ for an organization or a cultural practice that is valued—this is, indeed, exactly how we defined the institution. But what is valued by many, may be highly controversial to others. What is valued in one society is anathema in another. An organization revered in a certain era, may today be discussed as an example of malpractice or organized evil.

 Selznick’s three criteria do not resolve this normative conundrum. Take, for instance, the Federal Bureau of Investigations (FBI). When applied in a broad-brush, across-lifespan fashion, Selznick’s criteria would have us regard it as an institution. The FBI has a distinct identity: most people (not just Americans) have heard of the FBI and will have an idea of what it represents. The FBI has a unique competence and has existed for a long time. At the same time, it is easy to unearth a range of questionable values it espoused and activities it deployed for extended periods of its existence. For example, the latter decades of long-serving and founding Director J. Edgar Hoover were marked by practices that are now widely recognized as questionable if not outright illegal (Jeffreys-Jones 2007).

So what does this mean for Selznick’s criteria? What does it mean if organizations with dubious identities and questionable competences qualify as an institution? Should we reject these criteria and look for others? Should we avoid institutions that today are widely viewed as epitomizing questionable values?

We feel that Selznick’s criteria can still be used, but their use needs to be directed and qualified by situating organizations in a particular window of time and then assessing to what extent the institution embodied, advanced or provided stewardship of values deemed important by the society in which it existed. We must take into consideration that the value sets that stakeholders and the community at large apply to an organization can and do change over time. Institutions, in other words, are to be taken as organizations that have become effective and legitimate ‘guardians of public value’ *in a certain time and context*. When removed from that time and context, certain institutions or certain epochs in their existences or certain practices in which they engaged may well be considered dangerous or deplorable. The intriguing question is how such morally problematic organizations could maintain high levels of internal and external legitimacy at the time (cf. Selznick 1952).

If we suspend judgement, we can learn—even from institutions that in our time and context may look questionable—valuable lessons about their emergence, their value proposition, their governance, their ‘formula’ for success, their ways of acquiring a public licence to operate, their ways of navigating conflict and tensions, and in some instance, their decline and downfall. An institution tells us something about the society it emerges from and exists in.

This is also true for the cases included in this book. The fact that these particular organizations have ended up as specimens of ‘guardians of public value’ does not mean that we hold them up as being exemplary all of the time and in each respect. Most institutions go through periods of mission ambiguity and conflict; they are sometimes at a loss to develop distinctive competence and sustain effective practices, and they may have found it hard at times to adapt to significant changes in their context. Each case study will therefore situate the story of the institution in time and space, and treat its institutional (and normative) status as a variable and not as a given.

In this book, we study institutions that have done things and done them in ways that were of value to society and were indeed valued by their authorizing environments. Moreover, they have not merely ‘created public value’ (Moore 1995) and gained recognition for doing so, but continued to do so for considerable periods of time. They have acted not just as creators of public value, but as its guardians. Our aim is to introduce a set of sensitizing concepts in this opening chapter and see if they can help students, researchers and practitioners grasp and interpret the dynamics of institutionalization that have contributed to their lofty reputation and social status.

## Studying the Rise and Fall of Public Institutions

We are obviously not the first to study organizations as institutions. Selznick’s work inspired a series of detailed studies and theories that explain how organizations become institutions (DiIulio 1987; Wilson 1989; Boin and Goodin 2007; Boin and Christensen 2008; Goodsell 2011a, b) as well as accounts of how they become deinstitutionalized (Boin and ‘t Hart 2000; Alink et al. 2001; Collins 2009; Mair et al. 2014; Ansell et al. 2015). There is an entire library of institutional study material, both theoretical and empirical, in many different languages and in different disciplines. This research helps to explain why some public organizations (and not others) become—and remain—institutions. More specifically, it helps to answer three big questions that institutional scholars have endeavoured to answer.

- How do institutions emerge?

What are the drivers of institutionalization? Under what conditions does institutionalization happen? Do organizational characteristics matter? Does it matter in which ‘niche’ the organization operates? Is institutionalization an outcome of leadership? Or funding? Particular environmental demands? Happenstance perhaps?

- How do institutions stay strong and relevant?

Institutions have to perform a balancing act: distinctive (and cherished) competences must be wielded to satisfy societal expectations and perceptions of their performance. But societal expectations and perceptions of public organizations rarely remain fixed over long periods of time. This simple observation means that institutions are always vulnerable to contextual changes. They must, in other words, adapt to remain an institution—they must remain attuned to changes in both their operating environment (e.g. technological innovations, new products and services, competing organizations) as well as their authorizing environment (e.g. political power structures and governing philosophies). To adapt is also to risk alienation from societal expectations and perceptions. Becoming an institution may be one thing, remaining an institution is quite another challenge.

- What explains their decline and downfall?

Many long-standing institutions have ‘lost it’ and declined into oblivion. Whether we think of the Roman Senate or the Dutch East India Company, it’s clear that these were institutions in their time, remained institutions for a long time, and no longer exist. That prompts the question why institutions lose it. Do they somehow lose their capacity to adapt to changing expectations? Do their leaders succumb to hubris, dragging the institutions away from society? Or are these institutions faced with shocks that are so large and sudden that timely adaptation is simply impossible?

### Schools of thought

These three research questions have been studied by different theoretical traditions of institutional analysis that have been extensively described, summarized and compared elsewhere (Peters 2011; Scott 2013). To give an impression of the variety in explanations offered by students of institutions, let us just mention four dominant schools here.

 Philip Selznick was a pioneer of what is called *Classic Institutionalism*. This school focuses on organizations (as pillars of what they called the Organizational Society), seeking explanations for their emergence, functioning, effects and survival (see f.i. Thompson 1967; Wilson 1989). As we have seen, Selznick famously explained how and why institutions differ from ‘mere’ organizations. This happens when the organization is deeply valued by its employees, stakeholders, political leaders and the public. In explaining the emergence and downfall of these institutions, this approach pays a lot of attention to organizational leaders and the strategies they employ to maintain a relation between their organization and its environment.

Research in the tradition of *New Institutionalism* shifted attention from the individual organization to a class or type of organizations (Meyer and Rowan 1977; Deephouse and Suchman 2008). Where Selznick might focus on Oxford University as an institution, New Institutionalists would focus on the University (sui generis) as an institution. This School has done much to conceptualize the political and social environment in which particular types of organizations do or don’t evolve into institutions. The key idea is that organizations assume institutional properties by adhering to an ‘ideal-type’ that reflects how a society thinks about that type of organization. Through processes of ‘isomorphism’ these organizations are thought to adopt the required characteristics without necessarily changing the way they conduct their core business. While the New Institutionalists revived a scholarly interest in institutions, this school of thought has less interest in our core question: why do *particular* organizations become and remain institutions whereas others flounder and perish?

In recent years, a small group of political scientists became interested in the survival chances of public organizations. Inspired by the path-breaking work of Herbert Kaufman (1976), David Lewis (2002, 2004) built a database of US public agencies to test a theory that predicted ‘survivors’ would have different birth characteristics than non-survivors. A key assumption is that ‘normal’ organizations are perennially prone to capture, politicization and restructuring (Carpenter 2010). They can only survive these pressures, or so the theory goes, if they are ‘hardwired’ against efforts to terminate or co-opt the organization. The premise of this *Design School* has found empirical support: birth characteristics do seem to matter, as they raise survival *chances*. But they cannot explain which *particular* organizations will survive and which will perish.

Subsequent research has shown that design factors can only explain so much. *Population ecology* scholarship, for instance, also seeks to explain why some public organizations survive over time whereas others fold (Kaufman 1985; Boin et al. 2017; Kuipers et al. 2017; Van Witteloostuijn et al. 2018). Ecological studies are highly focused on structural and environmental factors. These studies offer support for the Design School, but also suggest that there are other factors at work at the population level. Their studies reliably show that the ‘carrying capacity’ of a population is probably the most important factor in predicting survival chances.

Both the Design School and the population ecologists tend to de-emphasize the potential role of behavioural and cultural factors in explaining institutionalization. Precisely those factors emerge in case studies of organizational and entrepreneurial success in both the public and corporate world (Lewis 1980; Peters and Waterman 1982; Doig and Hargrove 1990; Collins 2001; Malone and Fiske 2013). Also, there are many organizational biographies that describe the genesis, purpose and inner working of public and private institutions, offering in-depth accounts of their performance, legitimacy and endurance (Kaufman 1960; Boin 2001; Wetterberg 2009; Carpenter 2010). These biographies are not designed to draw general lessons or make comparisons. But they make clear that political and organizational leaders can affect the course of institutionalization.

## The Analytical Approach of This Volume

We began this project with a set of ideas that may explain why and how some organizations with a public purpose or public relevance end up acquiring *mission mystique* and become widely viewed as guardians of public value (and thus also why other organizations do not achieve this).

First, we don’t think institutions materialize by happy circumstance—they have to be created, maintained and protected. That requires a form of leadership, both within the organization and in its authorizing environment. At the same time, we do not believe that, as the Design School implies, institutions are ‘created by blueprint’. Institutions arise from organizations because they have the capacity to adapt and bounce back from the inevitable crisis. This requires a culture that is conducive to experimenting and learning (De Geus 1997; Goodsell 2011a, b).

Second, we are convinced that a public organization cannot do without some minimal level of legitimacy (Suchman 1995). Organizations become institutions because they *enjoy high levels of legitimacy for long periods of time*. While legitimacy is granted (or not) by those whose views count, organizations can actively work to earn that support. They can systematically regain it when it is has been compromised or lost; if these efforts are successful the organization *re*-*institutionalizes*.

In summary, we see an important relation between leadership and the process of institutionalization. We view institutionalization as a process that is at least partially spontaneous and unplanned. Organizations are not designed with a goal in mind to become an institution. But that does not mean that institutionalization simply happens, as a resultant of favourable circumstances or a dose of luck.

Following Selznick, we view institutionalization as an evolutionary process, which can be influenced but not fully controlled by organizational elites (Boin and Christensen 2008). Leadership is important as it guides, facilitates and shapes the process of becoming an institution. It is also critical for protecting the institution against the ‘forces of fragmentation’ (Kaufman 1960).

We conceptualize leadership as a collective endeavour by organizational elites to fulfil a set of tasks (cf. ‘t Hart and Tummers 2019). Leadership is not the property of the one individual who happens to occupy the highest rank in the organization. This helps us to escape from simplistic assumptions that institutional success is related to a particular leader (even if that leader stars in the organization’s mythology). At the other end of the continuum, we must be careful to relate lapses and pathologies of leadership directly to processes of deinstitutionalization (cf. Padilla et al. 2007; Helms 2012).

In summary, we follow Selznick in assuming that organizational leaders can guide the process of institutionalization in three ways (Selznick speaks of three executive tasks).

### Task 1: Shape the Identity of the Organization

 A key challenge for any public organization is that it must deliver on its formal (legal) assignment (or policy goals) while serving societal values and aspirations. If the organization solely seeks to deliver what it is built to deliver, the organization can quickly become redundant upon completion of the mission or shifting policy priorities. Organizations become institutions when they are perceived to embody societal ambitions *while* delivering on formal aims. To combine both is no easy task and will require tough choices—in a world of scarce resources, diverging preferences and bounded rationality, more often than not *something* will have to give. Making these choices amounts to a process of character building, which shapes the identity of the budding institution. Leaders can help shape this process, by facilitating experimentation that helps to discover the organization’s identity and by making critical decisions (Boin and Christensen 2008). Leaders also play a key role in communicating a sense of purpose, which keeps the organization aligned, determined and hungry.

### Task 2: Build and Nurture a Workforce That Can Deliver (and Loves to Do That)

An organization with a mission needs people ( professionals) who fit what the organization is trying to do. Selznick made an important point: professionals have to buy into the mission and believe in the underlying values that anchor the institution (cf. Kaufman 1960). This is especially true for public institutions, in which profit and competition cannot be the motivational drivers of the enterprise. Leaders of public institutions need to evoke and harness the ‘public service motivation’ of their employees (Perry and Hondeghem 2008). When professionals identify with and are energized by the mission, the management is relieved of the burdensome task of command-and-control duties. A high level of decentralization is then possible, as coordination is achieved through shared values (that functions as a ‘software of the mind’). The acid test is employees who proudly talk about their work and institution during birthday parties (cf. Dilulio 1994).

### Task 3: Preserve a Strong Relation with the Authorizing Environment

Institutions, by definition, enjoy a high level of legitimacy. They are valued by their stakeholders and, as often is the case, by society at large. It is a task of institutional leaders to protect and strengthen that relation. In doing so, leaders will face an inherent dilemma that will have to be negotiated time and again. Institutions enjoy a high level of legitimacy because they perform a task in an effective, consistent and highly valued way. Successful institutions are therefore not inclined to change their practices (which have been proven to work). But societal ambitions and preferences change. However well an institution may perform, they will be confronted with a ‘performance deficit’—the gap between societal expectations and perceived performance—sooner or later. If the deficit becomes too wide, an institution faces declining trust and may experience what we have labelled an institutional crisis (Boin and ‘t Hart 2000). Continuous adaptation of mission and work practices is therefore necessary. But such adaptation can rock the internal balance—the shared professional pride—that gives rise to its performance. When an institution changes slowly, it may be forced into reform; when it changes too quickly, employees may rebel. It is a leadership task to preserve a sensible balance.

## A Catalogue of Institutions: Introducing the Case Studies

This volume brings together case studies of very different organizations that managed to become institutions and have maintained their institutional status in the face of pivotal challenges, controversies and crises. A multidisciplinary cast of subject matter experts, guided by a shared analytical framework, provide educators and students with a rich array of teachable case studies.

The starting assumption underlying this volume is that many factors can shape the trajectory of institutionalization. Birth characteristics likely matter, as do the circumstances in which the institution saw the light. Leadership matters, both within and around the institution. Other institutions may cast their shadow. The same is true for historical contingencies. In short, we do not think it makes sense to hew closely to one particular school of thought, entering into a shadow boxing match with other theoretical schools. It is in this vein that we ‘instructed’ our authors: we gave them the freedom to identify and analyse factors that seemed to matter most in their individual case—leaving the door open for answers that we have not heard before.

Each chapter describes the story of an institution: its origins and early years; how it coped with change, adversity and crisis; the role of design, choice, chance and learning in these institutional trajectories. Each chapter has something to say about institutional leadership, in particular its balancing act of aligning mission, capacity and support in the face of ever-changing environments.

We selected cases that we could reasonably expect to have institutional characteristics (for at least a considerable part of their life spans to date). We also set out to include a wide variety of organizations. Our cases cover:

- *governmental and non*-*profit organizations* from a variety of countries and regions. Most cases are situated in the Western world, but there are also cases from Singapore and India.
- *very old and relatively young organizations*, ranging from the Swedish national bank (1668) to the World Anti-Doping Agency (WADA) (1999).
- organizations operating at *different levels of aggregation*—varying between locally grounded cases such as Singapore’s anti-corruption watchdog and Amsterdam’s Concertgebouw Orchestra to global players such as the Intergovernmental Panel on Climate Change (IPCC) and WADA.
- organizations performing *different kinds of public functions*—from production of cultural artefacts (such as BBC and Concertgebouw Orchestra), scientific knowledge (IPCC, CERN) and public infrastructures (Rijkswaterstaat) to delivering medical aid in complex emergencies (Doctors Without Borders), adjudicating disputes within or about the governance of the European Union (European Court of Justice), exercising regulatory oversight (ACCC, WADA) and enforcing the law ( Singapore’s Corrupt Practices Bureau).

We selected these cases for pedagogical and not for theory-building or hypothesis-testing purposes. We chose this variety of cases to give instructors and students alike a menu for choice. Cases were selected to allow readers to compare two or more most-similar cases, most-different cases, or other clusters of like/unlike characteristics. Readers should be able to draw on cases to identify patterns or perform plausibility probes on theoretical claims about institutions and institutionalization. Moreover, readers should be able to examine the impact of factors such as institutional contexts, organizational capabilities and institution-building leadership strategies.

The authors have been selected because they are experts on ‘their’ institution, not because they subscribe to our analytical framework. Each chapter loosely works around the framework set out above and thus actively encourages the reader to interpret the dynamics of each case. What actors, factors and mechanisms shaped the fate of the institution? What can we learn from this particular institutional history?

The volume offers case histories of the following organizations:

The *Indian Electoral Commission* has stewarded free and honest elections in the most populous and complex democracy on earth. The Commission successfully manages more than 1.4 million voting machines, 930,000 voting centres, 1.1 million government and 5.5 million civilian election employees, and more than half a billion voters. The story of its institutional development is one of mandate expansion: institutional leaders using the legal system to enhance the powers it can wield during election time.

*Singapore’s Corrupt Practices Investigations* *Bureau* was established in 1952 to battle the rife corruption present in all sectors of the public service, where bribes, favours and nepotism were fundamental norms of ‘doing business’. The Bureau had to earn its stripes fighting corruption among the country’s most powerful individuals: both in the police force and in the parliament. Through a record of successful actions against corrupt individuals, the agency gradually developed substantial authority to investigate any case in which corruption may be involved. The Bureau has been a driving force in making Singapore one of the least corrupt nations on earth.

The *British Broadcasting* *Corporation* (BBC) is one of Great Britain’s most venerable public institutions. It is also the world’s oldest ( created in 1922) and largest national public broadcaster (in terms of employees as well as its global reach and authority). Through its coverage, the BBC has documented and shaped the transformation of British society. It has maintained a reputation for impartiality and journalistic integrity. It has successfully weathered challenges, incidents and crises and continues to define the standard for quality broadcasting.

Celebrating its 350-year anniversary in 2018, the Swedish *Riksbanken* is the oldest central bank in the world. Its independence from government waxed and waned over time, and finally became firmly cemented in 1999. Its public authority helped Sweden survive the terrible 1992 economic crisis. In response to the global financial crisis that started in 2008, it was the first central bank to adopt negative interest rates to stimulate economic activity. In 2018, it announced that it was considering issuing E-Krona, electronic currency (the first central bank to do so).

The *European Court of Justice* (ECJ) is the undisputed guardian of the European Union’s transnational legal order, issuing landmark rulings and generating jurisprudence at a considerable pace. It was created in 1952 and has the power to invalidate the laws of EU member states when those laws conflict with EU law. The ECJ serves as the final arbiter of the growing body of international law that has accompanied the economic and political integration of Europe. It always faces the challenge of maintaining its authority and legitimacy when the EU’s ideals and institutions come under pressure.

The *Amsterdam Concertgebouw* *Orchestra* has historically been rated as one of the top symphonic orchestras in the world. Founded in 1888, it has had only seven chief conductors. It has found ways to balance the twinned but often conflicting imperatives of artistic excellence and financial viability. It remains dependent on government funding, which in the early 2010s became highly uncertain, triggering a mood of crisis that required astute management.

The *World Anti*-*Doping* *Agency* (WADA) promotes, coordinates and monitors the fight against performance-enhancing drugs in sports. WADA’s key activities include scientific research, education, development of anti-doping capacities and monitoring of the World Anti-Doping Code. Among the youngest organization in our set of cases, it came into being in 1999 as an independent foundation with a hybrid public– private structure. It is the chief guardian of the World Anti-Doping Code that has more than 600 signatories, including many states as well as international sports foundations. Its work has gained global recognition as contributing significantly to key values in sports such as fair play and the protection of athletes’ health and well-being.

 Founded in 1954 and based in Geneva, the *European Organization for Nuclear Research*, better known as CERN, is a remarkable example of enduring international scientific cooperation in pursuit of one of the most elusive goals ever embraced by any organization anywhere, one that requires sustained and large amounts of public funding. Among its key accomplishments are the pioneering of Internet technology, the creation of the World Wide Web, several Nobel Prize-winning staff members and the 2012 discovery of the Higgs Boson particle.

 Founded in 1798 during the French occupation, *Rijkswaterstaat*, the Directorate-General for Public Works and Water Management of the Netherlands, has evolved into an iconic institution. Its defining accomplishments are in the area of water management—digging canals, building and maintaining dikes, reclaiming vast tracts of land from the sea (‘polders’). Its planning and engineering feats are essential to the survival of a country where more than 25% of its territory lies below sea level and another 30% is highly exposed to flooding. The institution faced major adaptive challenges when its ‘safety-first’ paradigm was challenged by the rise of environmentalism. As it strives to transform itself, climate change is presenting another key test of its resilience, ingenuity and collaborative capacity.

Few humanitarian aid organizations enjoy a global public standing like *Médecins Sans Frontières* (MSF) has. Described as the most important humanitarian organization and conscience of the humanitarian world, MSF was awarded the Nobel Peace Prize in 1999. It displays a fierce sense of independence. Throughout its history MSF has often acted controversially, going public with its knowledge about atrocities committed by parties to the violent conflicts in which it operates, as well as explicitly challenging the humanitarian sector’s own practices and principles.

The *Intergovernmental Panel on Climate Change* (IPCC),  founded 1988, is the international body that reviews the latest science and produces assessment reports which inform international negotiations on climate change. It is tasked with establishing a consensus between climate experts and governments, communicating knowledge on climate change to policymakers, negotiators and the public, and for making recommendations on potential courses of action. The IPCC and Al Gore were co-recipients of the 2007 Nobel Peace Prize. The IPCC has also been criticized at times for its work and is a target of climate sceptics. The IPCC’s reputation was damaged when its leadership failed to respond effectively to mistakes found in its 2007 report. Since then it has worked to restore confidence. Its reports have been very important in the UN climate negotiations and strongly influenced the goals of the 2015 Paris Agreement.

Within a decade of its inception in 1995 the *Australian Competition and Consumer Commission* (ACCC) had become a trusted institution in the Australian regulatory landscape. The product of a merger, the ACCC soon carved out and dramatized its mission as a crusader for level-playing fields and fair play in markets. By successfully taking on some of the biggest corporations in the country in both the courtroom and the court of public opinion soon after coming into existence, the new authority quickly gained notoriety. By asserting its independence from political interference, it gained public credibility in a country that has long held its political class in low esteem. The ACCC has subsequently conserved its enforcement mission by adapting to challenges in the political and business environments, expanding and redirecting its repertoire for regulatory action, and broadening its consumer and small business constituencies.

## What Do the Cases Tell Us About the Dynamics of Institutions?

This book revolves around a central puzzle: Why do some public organizations become—and remain—institutions? Our relatively small and purposefully skewed set of case studies does not allow us to systematically test hypotheses, nor to generalize insights to larger populations of organization types. That said, what we can do is inductively identify possible patterns and relate them to conventional wisdom in academic theorizing and the world of practice. In interpreting these patterns, we can discern possible scope conditions or social mechanisms that may be at play in bringing about the institutionalization (and deinstitutionalization) of public organizations. More specifically, our cases provide food for thought with regard to four often-mentioned patterns of institutionalization. To help readers interpret the case studies, we will now discuss our observations in more detail.

### Pattern 1: Virtuous Cycles

**Fig. 1.2:**
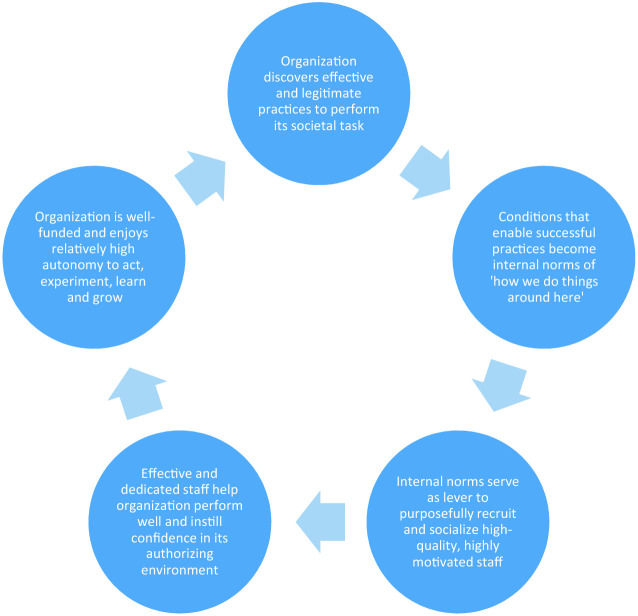
The virtuous cycle of institutionalization

In his study of highly successful corporate organizations, Jim Collins (2001, 2019) found that these organizations had one critical characteristic in common: they have in place what Collins refers to as a *flywheel*. Collins is, in essence, talking about what across the social sciences has been called a virtuous cycle: a set of processes that reinforce one another (Sitkin 1992; Finnemore and Sikkink 1998; Boin and Christensen 2008). Within an institution, the virtuous cycle might look like this (see Fig. 1.2):

- The cycle starts with the discovery or invention of an effective, efficient and legitimate way to reconcile organizational aims with societal aspirations. This typically happens through a mix of experimentation and smart copying.
- Successful practices give rise to the emergence of an internal norm: this is how we do things around here.
- The internal norm makes it easier to recruit and train the right people, which facilitates cohesion and effectiveness.
- Effective and dedicated people make the organization look good. This results in enhanced funding, support for the mission and strengthened autonomy.
- A strong and legitimate organization performs better, which solidifies the internal norm.

Our case studies bear witness to this virtuous cycle of institutions. Consider the following three examples:

The *Intergovernmental Panel on Climate Change* (IPCC) began as an informal collaboration of scientists who worried about climate change. By establishing a network of committed and reputable scientists, they created a platform for policymakers to learn about causes and potential solutions. The IPCC established ‘input and output legitimacy of the rigorous and extensive process by which the IPCC’s teams of expert authors and peer reviewers carry out their work’ (Paglia and Parker, this volume), which increased its epistemic power and reputation. Their reports helped to spread awareness about ongoing climate change, which, in turn, led to increased demand for evidence-based science. The growing interest of policymakers (prompted by growing awareness about the threat) helped to mobilize scientists who recognized a podium for their research. The density of scientific expertise secured privileged access to policymakers, which enhanced the importance of the IPCC.

Singapore’s *Corrupt Practices Investigations* *Bureau* (CPIB) began as a small police unit seeking to root out corruption among colleagues. When it busted a drug ring that was run by the police, the CPIB became an independent statutory authority. Its autonomy enhanced its investigative powers, which were widely and effectively applied. The success of the CPIB came to define Singapore’s status as a ‘clean’ state. Singapore’s enhanced international standing reflected back on the CPIB, which saw its autonomy and authority strengthened. Decades of successful investigations and prosecutions have embedded the institution in Singapore’s landscape (and indeed in the esteem of the international community). The CPIB’s effectiveness and Singapore’s reputation went hand in hand, reinforcing each other over the decades.

The *World Anti*-*Doping* *Agency* (WADA) was created to address the protracted doping crisis of the 1990s. Its chances of success seemed low. But it soon began to command the respect of its stakeholders, initially by formulating standards that made sense. As governments and sports foundations began to accept the standards, they also legitimated the Agency. As the Agency gained in stature, it could enlarge its role in the global fight against doping. The enlarged role translated into visible successes, which further strengthened its reputation. Quite incredibly, the WADA managed to become an undisputed authority in the international field of sports. Its role in other sports organizations became entrenched, which further helped the standards to take root.

### Pattern 2: Institution-Building Leadership

 Institutionalization does not just happen; virtuous cycles do not simply materialize. This prompts the question if and to what extent the actions of leaders matter when it comes to the institutionalization process. The chapters in this book do not give rise to a new or definite take on this critical question. But they certainly provide powerful illustrations of leaders forging practices, crafting norms and protecting the identity and integrity of their organizations—and they show that this can be done in different leadership configurations and employing different leadership styles. Let’s look at some examples to illustrate this variety:

The institutional history of the *Amsterdam Concertgebouw* *Orchestra* cannot be written without recognizing its early and long-serving conductor Willem Mengelberg. He was the archetypical institution builder, translating the aspirations of the founding regents into an ambitious and appealing musical vision for the orchestra. Mengelberg then translated this vision into an unprecedented and uncompromising regime of excellence that produced both classical and contemporary symphonic music, while building an international audience. By placing his orchestra squarely on the map, Mengelberg forged a broader authorizing environment for his orchestra, extending well beyond the original group of regents.

The *European Court of Justice* (ECJ) began as a technical tribunal. It was hard to imagine at the time how this small court in Luxembourg could become an institution, creating conditions that today make European integration a seemingly one-way road. This did not happen because of one leader. It happened because a group of judges—all appointed sometime in the early 1960s—shared a vision and began to build the ECJ in light of that vision. These judges were well-known professionals who moved in the insulated elites that pushed for European integration. Without seeking the limelight, they exerted the leadership of true institution builders.

The founding Commissioner of the *Australian Competition and Consumer Commission*, Allan Fels, not only brought academic expertise and long regulatory experience to the job, but also a brisk determination to give the new agency the institutional clout its predecessors had often lacked. Painstakingly independent and politically neutral, Fels used the media to create a powerful platform for the ACCC’s ‘naming and shaming’ of big corporations that engaged in anticompetitive or manipulative behaviour. In prosecuting and winning high-profile cases, he instilled professional pride in its staff and ensured the ACCC became a highly visible and impactful crusader for consumers.

### Pattern 3: Mature Management of Conflict

 Institution building is more than formulating an evocative mission. Professionals must be seduced and coaxed to accomplish the mission (leaders cannot do it by themselves). This can be an arduous job, as the chapters suggest. Institutions are not, by definition, happy families (certainly not all the time). A public institution must find ways to harness conflict in ways that make it smarter and stronger (Coser 1956). The chapters show how institutions do not always suppress conflict, but manage to canalize it.

The *European Organization for Nuclear Research* ( CERN) did not become a celebrated scientific institution without tension or strife. Bringing together the best scientists in the world and have them compete for funds can be a recipe for disaster. CERN developed a form of shared leadership, which allowed this international community of super-smart scientists to evolve ‘ norms and practices of balance-seeking’:

Balance between funding member states and the spending CERN administrators. Balance between small and large contributors. Balance between centralized lab and infrastructure funding and bottom-up funding of the experiments. Balance between getting on with current work and preparing the ground for taking on new challenges and realizing future ambitions that are decades away. Balance between the scientists’ advances in fundamental physics and the engineers’ development of the technological tools required to test them. Balance between running a tight ship financially and maintaining the ability to respond flexibly to financial setbacks or emerging expenditures. Balance between the patience required to do the work necessary to achieve major scientific breakthroughs and the need to be seen to be active, relevant and impactful in the present vital to maintain the institution’s global public and political support base. Balance between banking on the authority of established scientific leaders and on empowering the innovative irreverence of emerging research talents. (Engelen and ‘t Hart, this volume)

The governance of the *Concertgebouw Orchestra* has been marked by decades of tension between protagonists of its artistic aspirations and business managers seeking to ensure the organization remained financially viable. It describes how Mengelberg, the legendary conductor, waged no-holds-barred battles with a succession of business managers and artistic directors who had the temerity of proposing pragmatic rather than ‘perfect’ options to address pressing financial challenges. It references the painful, unnecessary and politically costly estrangement of maestro Bernard Haitink from the orchestra during the latter years of his highly successful tenure. But the story also demonstrates, using Coser’s (1956) words, the positive functions of social conflict: the many conflicts resulted in a change of the governance model, which finally resolved the long-simmering tensions between artistic excellence and financial viability.

The birth of *Médicins Sans Frontières* was rooted in a conflict of values and criticism of the status quo in mainstream humanitarian aid organizations such as the Red Cross. Witnessing severe atrocities among civilians during the Nigerian civil war in the late 1960s, doctors were forced to remain silent under the Red Cross’s principle of ‘ neutrality’. This motivated a group of French doctors to set up MSF as a breakaway organization. It set the organization on a path of fierce independence, going public with inconvenient truths and occasionally engaging in very public withdrawals from theatres of conflict where the integrity of its operations was being undermined by conflicting parties. Its contrarian ethos also affected MSF’s internal culture: its policies and strategies took shape though sometimes sharp disagreements about the right thing to do in war-torn areas.

### Pattern 4: Adaptive Capacity

Organizations become institutions because they somehow maintain high performance over the course of their existence. Institutions have survived many cultural, societal and political contexts changes. Institutions face constant threats to its engrained and established formula, yet manage to preserve their virtuous cycle. This requires timely, in some cases even pre-emptive, forms of adaptation to maintain the flywheel.^1^

Our case studies suggest how institutions manage to accomplish this. Institutions monitor the environment for new demands and potential threats; they probe the internal culture for complacency and newly emerging fault lines that have the potential to compromise the institution’s integrity and performance. Institutions maintain a culture of learning, innovation and contestation—they are ‘charged with vitality’ (Goodsell 2011a).

*Rijkswaterstaat* provides a fascinating case study in this regard. The traditional institution was at first reluctant to acknowledge that its technocratic paradigm of project planning and management had gone past its sell-by date as a result of changes taking place in Dutch society during the 1960s. But once its eyes were opened, it went on a learning journey that continues into the present. Rijkswaterstaat keeps trying to reconcile the traditional strength of ‘go-it-alone civic engineering’ with the ‘soft skills’ and ‘ collaborative mindset’ required to thrive in a post-paternalistic era.

The *European Court of Justice* is another intriguing example of adaptive capacity. Just when the Court had found its institutional footing and delivered hallmark rulings that would cement European integration for decades to come, the European project itself came under intense criticism (in the 1970s). Around that time, the most influential judges in the Court were set to retire. This confluence of events created a dire need to revisit and rethink the way the Court functioned.

This brings us to what is known as the *paradox of success*: the capacity to adapt can be undermined by the successes of the institution. The very strength of an institutional formula sows the seeds of the institution’s demise. The operative mechanisms here are not only the kind of hubris, complacence and rigidity foreseen by Selznick (1957) as chief forces of erosion of institutional integrity. There is something much more mundane at the heart of it: the dedicated adherence to what has been proven to work well makes it seemingly unnecessary to consider alternative ways of working that may be better suited for dealing with evolving contexts and new challenges.

**Fig. 1.3:**
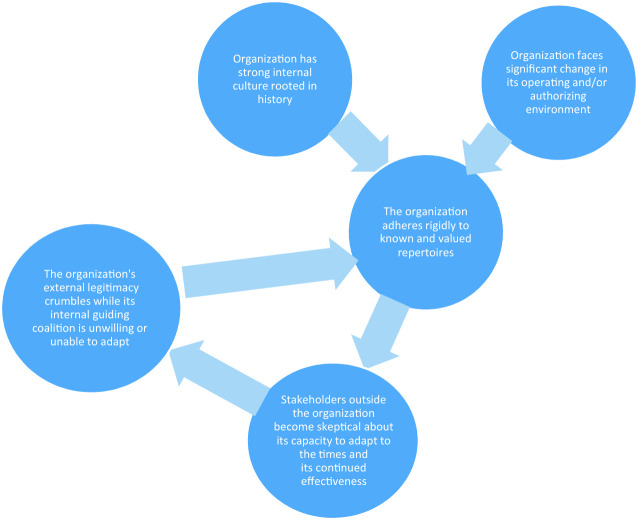
The vicious cycle of deinstitutionalization

Figure 1.3 captures how a virtuous cycle can turn into a vicious cycle of deinstitutionalization, which can be described as follows (Masuch 1985; Boin and ‘t Hart 2000; Ansell and Bartenberger 2017):

- Successful practices give rise to a strong internal culture (‘this is how we do things around here’) that makes it hard to suggest or even imagine alternative ways of working.
- Institutional members do not recognize impending threats to the institutional model.
- When shifting contexts and new challenges begin to undermine the effectiveness, efficiency or legitimacy of that very model, the institution doubles down on what it believes to be the best practices.
- This is perceived by parts of its authorizing environment as a refusal to acknowledge the need for change.
- Legitimacy declines as a result; criticism begins to mount.
- The institution is at a loss of what to do, falling back on practices that are still assumed to work (but actually exacerbate the problem).
- As perceptions of institutional performance continue to decline and institutions demonstrate limited or no willingness to change, conditions for an institutional crisis are create d .

If allowed to continue, this process of deinstitutionalization can create an existential crisis for an institution. It requires exceptional leadership to guide the institution through such a period. For example, the so-called Climategate crisis facing the *IPCC*, when inaccuracies in its Fourth Assessment Report were revealed, opened up the institution to charges of bias, hidden agendas and politicization of its processes and findings. Playing into the hands of ‘climate deniers’, the crisis put pressure on the IPCC to acknowledge its fallibility, which, in turn, appeared to confirm the criticism put forward by its critics. The IPCC survived the crisis by creating procedures that enhanced the integrity of its findings and conclusions.

Anti-doping watchdog *WADA* was plunged into an institutional crisis of its own making. WADA had failed to detect the brazen, systematic subversion of its norms and its compliance regime by the Russian sports federations, peaking at the Sochi Winter Olympics. Its initial response to whistleblowers, which came forward from within the Russian system, was inept. It also proved unable to orchestrate support for firm sanctions. The organization compounded its problems by gullibly declaring its Russian counterpart Rusada fully compliant again in 2018, a declaration it had to retract when it transpired that the data on which the decision was based had been tampered with. But the crisis did not undermine the belief that without an institution such as WADA there can be no credible anti-doping policy.

## The Future of Institutions in Turbulent Times

Institutions are conceived in this book as ‘societal safeguards’. Institutions protect our better angels from the detrimental effects of partisanship, moral panics, opportunism (the temptation of the quick fix) and adventurism (the temptation of the sweeping promise). Institutions, in other words, protect us from ourselves. This is especially important in a time when public entities are fragile to the whims of public opinion and the impulses of politicians. Some argue that today’s institutions experience unique challenges (Rosanvallon 2018).

 One challenge derives from the rise of what has been called ‘monitory democracy’ (Keane 2018). In monitory democracies, all forms of authority, all organizations and all power-holders are continuously vetted, interrogated and challenged by a plethora of accountability regimes. If institutions indeed depend on a certain ‘mystique’ in gaining and maintaining their elevated status, the question arises whether that mystique can survive the withering challenges of hyper-transparency, the relentless thickening of accountability and the growing vagaries of public emotions and political will (Hood 2010; Bovens et al. 2014; Busuioc and Lodge 2017).

The dense web of arrangements and rituals of verification and control has made institutional ‘ mission mystiques’ more fickle. Operating in today’s more polarized social and political climate may make it hard for institutions to claim that they are serving the ‘public interest’ (McCoy et al. 2018). Institutions can, in other words, rely less and less on their legal mandates, their formal autonomy and their professed commitment to certain fundamental values—to ensure their social licence to operate. Institutional legitimacy has become more dependent on whether they actually ‘perform’ (hitting targets, scoring wins) and how they go about performing (i.e. complying with ever more strongly worded and policed procedural norms of transparency, fairness and propriety).

 A second challenge is grounded in the shift from the machine age to the network age (Castells 1996). Twentieth-century organizing was grounded in hierarchy, specialization and compartmentalization of knowledge, funding, task performance and responsibilities. The dominant ideology of twenty-first-century organizing is one of collaborating across boundaries, pooling of resources, flexible arrangements, shared power and responsibility. In the face of relentless globalization, wicked problems and transboundary crises, public institutions find it hard to position themselves as islands or bulwarks organized around a self-proclaimed mission and accompanying values.

Today’s world is very different from the America of the mid-1950s where Selznick wrote his path-breaking book *Leadership in Administration*. Collaborative approaches to public innovation, societal problem-solving and dealing with wicked issues and creeping crises have become the new normal (Emerson and Nabatchi 2015). If they are to remain relevant in a network society, public institutions must be able to act as co-creators in hybrid public–private, intergovernmental and transnational collaborations. The question is how institutions can align or even coalesce their operations, norms and identities with network partners in service of a shared purpose without diluting their own institutional character. In modern society, the concept of a focal organization with a distinct mission, structure, value set, membership and value chain may have to be refreshed.

But it would be premature at best to announce the impending death of public institutions and the obsolescence of the kind of institutional analysis performed in this book. The virtuous cycles that these institutions create and maintain are needed perhaps even more than before. Networks may be important to make complex service delivery or emergency response work, but they don’t have the visibility and established identity that institutions enjoy. Citizens of monitorial democracies may have become more transactional and less forgiving in how they assess those organizations and their leaders. By the same token, however, in times of turbulence and confusion, citizens (and markets) look for beacons of hope, protection, direction and order. Public institutions may well be our best hope in times of turbulence.

Public institutions are guarding something that is probably older and weightier than the current interests and priorities of any one group or party in the system, however powerful. Whether in times of turbulence of relative stability, the pivotal challenge for institutional leaders remains threefold: to make the case for its *raison d’être* by claiming guardianship of salient, widely desired public values; to motivate people so that the institution delivers on its licence to operate; and to continuously adapt the organization’s make-up, beliefs and practices to remain in tune with the norms and demands of ever-changing times. These are no easy tasks. The chapters in this book provide tales and insights that will hopefully prove useful for future institutional leaders. To facilitate classroom use of each case study we have placed questions for discussion at the end of each chapter.

A final note. The manuscript of this volume was closed just weeks before the Covid-19 pandemic enveloped and disrupted practically the entire world, thus presenting people, businesses, community organizations, government and international organizations with an unprecedented ‘stress test’ of their adaptive capacity. It remains to a next group of researchers to investigate whether and how the institutions covered in this book managed to not only survive but productively absorb and adapt to the immense challenges the virus and its many impacts presented.

### Note

1. The kind of consolidating leadership that is required to ensure the institution stay relevant and valuable externally and continues to cohere internally has been captured nicely by Frederickson and Matkin (2007) in their essay on public leadership as ‘gardening’.
